# MLKL inhibits intestinal tumorigenesis by suppressing STAT3 signaling pathway

**DOI:** 10.7150/ijbs.56152

**Published:** 2021-02-17

**Authors:** Qun Zhao, Xinran Cheng, Jian Guo, Yun Bi, Li Kuang, Jianhua Ren, Jing Zhong, Longrui Pan, Xudong Zhang, Yang Guo, Yongqiang Liu, Shu Jin, Yan Tan, Xianjun Yu

**Affiliations:** 1 Laboratory of Inflammation and Molecular Pharmacology, Hubei Key Laboratory of Embryonic Stem Cell Research, School of Basic Medical Sciences & Biomedical Research Institute, Hubei University of Medicine, Shiyan 442000, China.; 2 Department of Oncology, Affiliated Dongfeng Hospital, Hubei University of Medicine, Shiyan 442000, China.; 3 State Key Laboratory of Molecular Oncology, National Cancer Center, National Clinical Research Center for Cancer, Cancer Hospital, Chinese Academy of Medical Sciences and Peking Union Medical College, Beijing 100021, China.; 4 Institute of Clinical Pharmacology, Guangzhou University of Chinese Medicine, Guangzhou 510405, China.; 5 Department of Gastroenterology, Taihe Hospital, Hubei University of Medicine, Shiyan 442000, China.

**Keywords:** Intestinal tumorigenesis, MLKL, IL-6/STAT3, Anti-IL-6R antibody therapy.

## Abstract

Mixed lineage kinase domain-like protein (MLKL) plays an important role in necroptosis, but the role and mechanism of MLKL in intestinal tumorigenesis remain unclear. Here, we found that hematopoietic- and nonhematopoietic-derived MLKL affected intestinal inflammation, but nonhematopoietic-derived MLKL primarily inhibited intestinal tumorigenesis. Loss of MLKL enhanced intestinal regeneration and the susceptibility to intestinal tumorigenesis in *Apc^min/+^* mice by hyperactivating the Janus kinase 2 (JAK2)/ signal transducer and activator of transcription 3 (STAT3) axis. Furthermore, MLKL deficiency increased interleukin-6 (IL-6) production in dendritic cells. Administration of anti-IL-6R antibody therapy reduced intestinal tumorigenesis in *Apc^min/+^Mlkl^-/-^* mice. Notably, low MLKL expression in human colorectal tumors, which enhanced STAT3 activation, was associated with decreased overall survival. Together, our results reveal that MLKL exhibits a suppressive effect during intestinal tumorigenesis by suppressing the IL-6/JAK2/STAT3 signals.

## Introduction

Colorectal cancer is the third most common cancer and is associated with high mortality worldwide 1. Various risk factors is associated with the initiation and progression of colorectal cancer, including genetic mutations, inflammation, gut microbiota and epigenetic modification 2. Although approximately 90% of patients can be cured at the early stage, colorectal cancer is usually detected at an advanced stage 3-4. Therefore, identify promising targets for predicting colorectal cancer risk and disease progression is needed.

Necroptosis is caspase-independent necrosis that requires the receptor-interacting protein kinase 1 (RIPK1) protein and RIPK3. RIPK1 and RIPK3 interacts via the RIP homotypic interaction motifs (RHIMs) and facilitates the formation of amyloid signaling complex RIPK1-RIPK3 necrosome during necroptosis. MLKL, an important downstream mediator, is activated by RIPK3 and then oligomers to disrupt the membrane integrity, leading to necroptotic cell death 5-7. Recent studies have shown that multiple key mediators of necroptosis are involved in the progression of tumors 8-9. *Ripk3*-deficient mice are susceptible to intestinal tumorigenesis 10-11. Notably, reduced MLKL expression in tumors is significantly correlated with poor overall survival 12. Recently, we reported that loss of MLKL accelerated colitis-associated tumorigenesis 13. However, the precise mechanisms and signaling pathway by which MLKL participates in intestinal tumorigenesis remain unclear. Notably, colitis-associated tumorigenesis represents only 1% of colorectal cancer, and sporadic intestinal tumors are more prevalent 14. Thus, it is necessary to elucidate the role and the underlying mechanisms of MLKL in sporadic intestinal tumorigenesis.

Signal transducer and activator of transcription 3 (STAT3) as a transcription factor that regulates a large number of physiological and pathological cellular processes 15-18. Hyperactivation of STAT3 plays an important role in numerous malignant tumors 19-20. Accumulating evidences have reported that persistent activation of STAT3 promotes colorectal cancer progression 18, 20. Interleukin-6 (IL-6), a prominent member of the IL-6 family of cytokines, forms a complex with signaling receptors 21. IL-6 binds to IL-6Rα and forms IL-6-IL-6Rα complexes, subsequently interacts with the membrane-associated gp130 subunit, leading to the phosphorylation of Janus kinase 2 (JAK2) 22. JAK2 activation promotes the phosphorylation, nuclear translocation, and transcriptional activation of STAT3 23.

In the present study, we revealed that loss of MLKL increased the susceptibility to intestinal regeneration and tumorigenesis through hyperactivation of IL-6/JAK2/STAT3 signaling. Notably, anti-IL-6R antibody therapy effectively suppressed intestinal tumorigenesis and STAT3 activation in *Apc^min/+^Mlkl*^-/-^ mice. We further found that reduced MLKL expression was correlated with poor prognosis and STAT3 hyperactivation in colorectal tumors. Thus, our findings emphasized that loss of MLKL in intestinal tumors exhibited aggressive disease.

## Materials and methods

### Animal experiments

*Mlkl^-/-^* mice were generated and genotyped as previously described 13. *Mlkl^-/-^* mice were crossed with *Apc^min/+^* mice to generate *Apc^min/+^Mlkl^+/-^* and *Mlkl^+/-^* mice. Then, *Apc^min/+^Mlkl^+/-^* mice were crossed with *Mlkl^+/-^* mice to generate *Apc^min/+^* and *Apc^min/+^Mlkl^-/-^* mice. All the mice were on a C57BL/6 background and maintained in a specific pathogen-free (SPF) facility. Animal experiments were performed according from Institutional Animal Care and Use Committee of the Hubei University of Medicine (approval no. 2019-066).

### AOM/DSS induced colorectal carcinogenesis

8-week-old mice were injected intraperitoneally with azoxymethane (AOM, 10 mg/kg). After 5 days, mice were treated 2.5% (m/v) dextran sodium sulfate (DSS) in normal drinking water for 7 days, and then administrated normal water for 14 days. The DSS-normal water treatment cycle was repeated three times. At the third cycle on day 80, the mice were euthanized and intestinal tumors were collected for further analysis.

### Bone marrow transplant

To address the function hematopoietic or/and nonhematopoietic cells to intestinal tumorigenesis, chimeric mice were generated as previously described 24-25. In brief, 6-week-old recipient WT mice or *Mlkl^-/-^* mice were lethally irradiated (9 Gy) to remove bone marrow cells, WT or *Mlkl^-/-^* recipient mice were injected with 1×10^7^ bone marrow cells via the tail vein from WT mice or *Mlkl^-/-^* donor mice. WT→WT, WT→*Mlkl^-/-^*, *Mlkl^-/-^*→WT and *Mlkl^-/-^*→*Mlkl^-/-^* chimeric mice were fed water supplemented with 2 g/L neomycin sulfate for two weeks. Chimeric mice were subjected to establish AOM/DSS-induced colorectal tumor models after eight weeks of bone marrow reconstitution.

### Determination of clinical scores

The scoring of stool consistency and occult blood was performed as previously described 26. The disease activity index (DAI) was determined based on the combined score.

### Tumor load

Tumor load was determined according to the published protocol 27. Tumor load was calculated as follows: count tumor numbers, measure tumor size, and calculate tumor load per mouse.

### Whole-body irradiation

Whole-body irradiation (WBI) was performed according to published protocol 28. Eight-week-old mice (n=5 *per* groups) were subjected to 10-Gy WBI. At 3 and 5 days, the intestinal was subjected to H&E staining, and the regenerating villi length was analyzed in 10 fields per mouse. The intestinal tissues were collected for further analysis.

### RNA-sequencing

Intestinal tissues were obtained and isolated RNA from 6-week-old *Apc^min/+^* and *Apc^min/+^Mlkl^-/-^*mice. The libraries of mRNA were conducted in Illumina HiSeq X-ten system (Shanghai Bohao Biotechnology). The raw reads were filtered and mapped to the genome by Seqtk and TopHat, respectively 29. Fragments gene were reassembled using HTSeq 30-31, and significant differentially expressed genes (DEGs) were clarified based on False Discovery Rate (FDR) (Q< 0.05) and fold-change >2 32.

### Quantitative reverse-transcriptase PCR (qRT-PCR)

Intestinal tissues were collected and homogenized in TRIzol reagent (Life Technologies) using a Mini-Rotor (Thermo Scientific). The transcript levels of genes were measured by qRT-PCR analysis.

### Western blotting

Isolated intestines were homogenized using a Mini-Rotor (Thermo Scientific). Tissues were lysed and protein concentrations were quantified. The proteins were separated by SDS-PAGE gels and were detected using chemiluminescent substrate (Thermo Scientific). Antibodies for western blotting used included: rabbit anti-MLKL (#ab184718, Abcam, 1:1000), rabbit anti-phosphor-STAT3 (Tyr 705) (#9145, Cell Signaling Technology, CST, 1:1000), mouse anti-STAT3 (#9139, CST, 1:1000), rabbit anti-PCNA (#13110, CST, 1:1000), rabbit anti-Cleaved-Caspase-3 (#9661, CST, 1:1000), rabbit anti-Cyclin D1 (#2978, CST, 1:1000), rabbit anti-C-myc (#9402, CST, 1:1000), and rabbit anti-GAPDH (#GB11002, Wuhan Service Bio Technology Co., Ltd., 1:5000).

### Intestinal tissue for the analysis of cytokine production

The intestinal tissues were homogenized and lysed. The lysates were centrifuged and the protein concentration was measured by ELISA (eBioscience) 33.

### Isolation of intestine epithelia cells (IECs) and lamina propria lymphocytes (LPLs)

Isolation of intestinal epithelial cells and lamina propria lymphocytes was performed as previously described 34. Intestines were cleared and then cut into pieces. The IECs were collected following shaking after incubation in cold PBS with 3 mM EDTA/1.5 mM DTT. The remaining tissues were further digested by collagenase type XI (Sigma) and DNase I (Sigma) at 37°C for 1 h, and the LPLs were isolated from the supernatants by Percoll. For IL-6 stimulation, intestinal tissues were digested by collagenase type I (Sigma) and Dispase II (Sigma) for 2 h. Cells were plated into 6-well plates coated with type I collagen (Invitrogen) overnight after washing at least three times.

### Anti-IL-6R antibody treatment

4-week-old *Apc^min/+^* and *Apc^min/+^Mlkl^-/-^*mice were intraperitoneal injected with 4 mg/kg tocilizumab (#A2012, Selleckchem; Houston, TX, USA) weekly. After 10 weeks of treatment, mice were euthanized, and the intestinal tumors were collected for further analysis.

### Clinical specimens

Tissue microarrays data on 98 colorectal cancer samples and 79 adjacent samples were obtained from (Shanghai Outdo Biotech Co., Ltd). An anti-MLKL antibody (#ab184718, Abcam; 1:100) was used to detect the expression of MLKL in the immunohistochemical assays. The samples were scanned to obtain high-resolution digital images. The expression of MLKL was scored and quantified by pathologists. The quantified multiplicative index was determined as follows: 0 to 3: the average staining intensity; 0 to 4: extent of staining in the scores. The low MLKL staining indexes ranged from 0 to 4, and the higher MLKL staining indexes ranged from 4 to 12. Above experiments was approved by the ethics committee of Hubei University of Medicine (Hubei, China) (approval no. 2019-TH-027).

### Statistical analysis

Data are expressed as the mean ± standard errors of the mean (SEM). Statistical significance was conduced using 2-tailed Student's *t* test, one-way ANOVA, log-rank test or Pearson's correlation coefficients test. *P*-values < 0.05 were considered statistically significant.

## Results

### MLKL in hematopoietic and nonhematopoietic cellular compartments mediates protection against intestinal tumorigenesis

**Figure 1:**
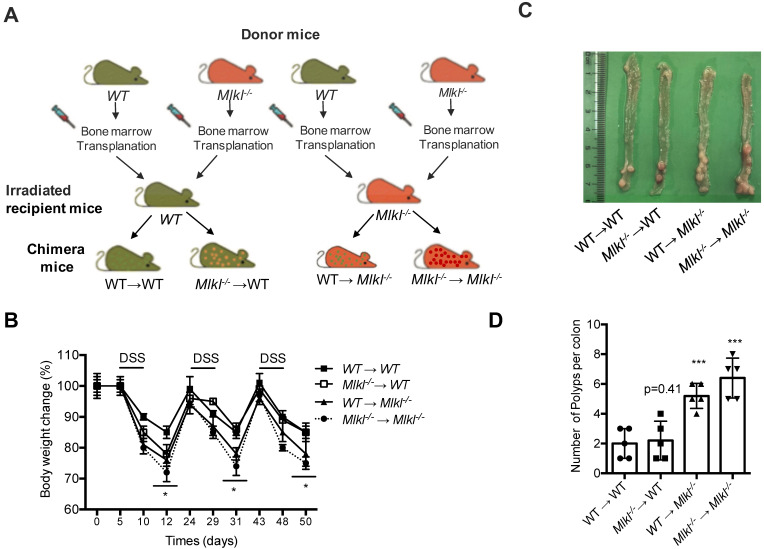
** MLKL in hematopoietic and nonhematopoietic cellular compartments mediates protection against intestinal tumorigenesis. (A)** Schematic overview of four chimeric mice: WT → WT, WT → *Mlkl^-/-^*, *Mlkl^-/-^*→ WT, and *Mlkl^-/-^* → *Mlkl^-/-^*. **(B)** Weight change in the chimeric mice of the AOM/DSS model. **(C)** Representative images of the intestinal tumors from chimeric mice on the day 80 of the AOM/DSS model. **(D)** Quantification of the polyp numbers in chimeric mice in the AOM/DSS model. * *p* < 0.05 and *** *p* < 0.001 *versus* WT → WT groups.

Dysplastic epithelial cells and myeloid cells are involved in intestinal tumorigenesis 35. MLKL is widely expressed in various cell types and organs as shown by others studies and our previous findings 6, 36-37. Thus, the function of MLKL in in hematopoietic and/or nonhematopoietic cellular compartments might be associated with intestinal tumorigenesis. To gather further evidence, we established four groups of chimeric mice (WT→WT, *Mlkl^-/-^* →WT, WT→*Mlkl^-/-^*, *Mlkl^-/-^*→*Mlkl^-/-^*) and were subjected to AOM/DSS-induced colorectal carcinogenesis **(Figure 1A)**. During the first cycle of DSS treatment, the WT→*Mlkl^-/-^* and *Mlkl^-/-^* →WT chimeric mice showed similarly weight loss relative to *Mlkl^-/-^*→*Mlkl^-/-^* mice **(Figure 1B)**, suggesting that MLKL in both hematopoietic and nonhematopoietic cellular compartments was necessary during this phase. During the second cycle of DSS, we found that the WT→*Mlkl^-/-^* mice, but not the *Mlkl^-/-^* →WT mice, showed more weight loss, suggesting that the effect of MLKL was mainly dependent on its expression in the nonhematopoietic cellular compartment during the later stage of intestinal tumorigenesis. In addition, clinical scores also confirmed these phenomenon (**Figure S1 and Table S1**). Correspondingly, the *Mlkl^-/-^*→*Mlkl^-/-^* mice exhibited more intestinal polyps than the WT→WT mice (**Figure 1C, 1D**). Moreover, the WT→*Mlkl^-/-^* mice had significantly more intestinal polyps than in WT→WT and *Mlkl^-/-^*→WT mice (**Figure 1C, 1D**). However, the number of intestinal polyps in the *Mlkl^-/-^*→WT mice was not significantly different from the WT→WT mice (**Figure 1C, 1D**). These results show an early role of MLKL in hematopoietic and nonhematopoietic cellular compartments and indicate that MLKL in nonhematopoietic cellular compartments is critical for the inhibition of intestinal polyp formation.

### MLKL deficiency increases intestinal tumor burden in the *Apc^min/+^* model

**Figure 2:**
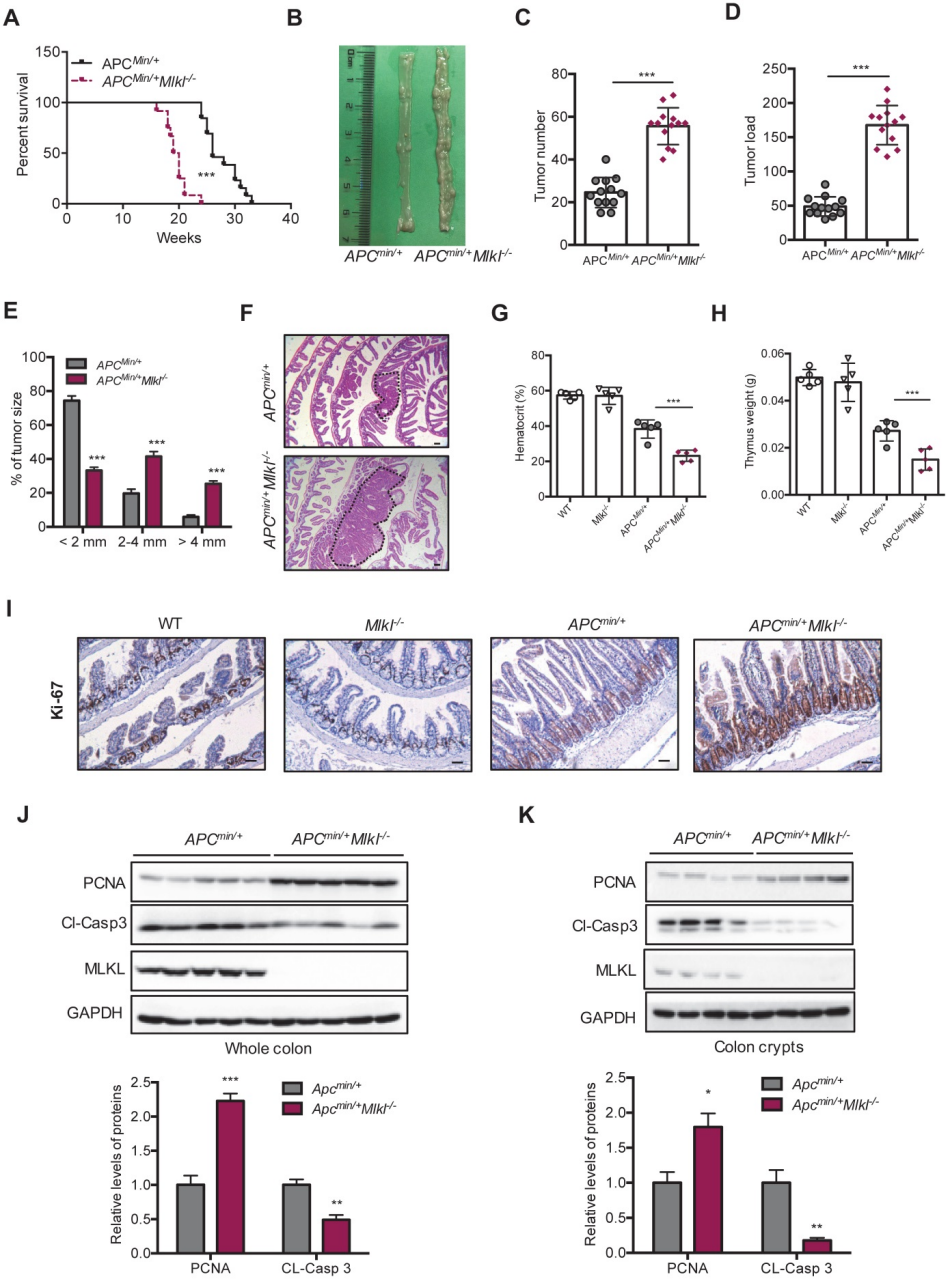
** MLKL deficiency increases intestinal tumor burden in the *Apc^min/+^* model. (A)** Survival of *Apc^min/+^* and *Apc^min/+^Mlkl^-/-^* mice. **(B)** Polyps in representative intestines from *Apc^min/+^* and *Apc^min/+^Mlkl^-/-^* mice. **(C and D)** Quantification of polyp formation (C) and tumor load (D) in 16-week-old *Apc^min/+^* and *Apc^min/+^Mlkl^-/-^* intestines. **(E)** Distribution of tumor size in 16-week-old *Apc^min/+^* and *Apc^min/+^Mlkl^-/-^* mice. **(F)** Images of the H&E-stained intestines from* Apc^min/+^* and *Apc^min/+^Mlkl^-/-^* mice. **(G and H)** Hematocrit (G) and thymus weight (H) of 16-week-old WT, *Mlkl^-/-^*, *Apc^min/+^* and *Apc^min/+^Mlkl^-/-^* mice. **(I)** Ki-67 staining of the representative intestines from age-matched WT, *Mlkl^-/-^*, *Apc^min/+^* and *Apc^min/+^Mlkl^-/-^* mice. (J-K) Whole intestines and colonic crypts were isolated from 16-week-old *Apc^min/+^*and *Apc^min/+^Mlkl^-/-^* mice. PCNA **(J)** and cleaved caspase-3 **(K)** were analyzed by western blotting. Scale bars: 50 μm. * *p* < 0.05, ** *p* < 0.01, *** *p* < 0.001 *versus Apc^min/+^* mice.

APC mutations are frequently found in intestinal tumors patients 38. *Apc^min/+^* mice develop spontaneous intestinal polyps and become a good animal model for investigating intestinal tumorigenesis 39. To unveil the function of MLKL in intestinal tumorigenesis, we analyzed tumor progression in *Apc^min/+^Mlkl^-/-^*mice and *Apc^min/+^* mice. The survival time was dramatically decreased in the *Apc^min/+^Mlkl^-/-^* mice, and the median survival of these mice was only 127 days relative to the median survival of the *Apc^min/+^* mice, which was 185 days **(Figure 2A)**. Macroscopic dissections showed increased lesions in the *Apc^min/+^Mlkl^-/-^* mice compared to the *Apc^min/+^*mice at 12 weeks of age (**Figure 2B**). However, there were no overt defects in *Mlkl^-/-^* intestines compared with those of the WT mice during the 12-months of observations (**Figure S2A**). Moreover, no significant difference in proliferation and self-renewal was found in WT and *Mlkl^-/-^* intestines (**Figure S2B**), suggesting that MLKL was only functional under disease conditions. In addition, the *Apc^min/+^Mlkl^-/-^*mice had markedly increased tumor numbers (**Figure 2C**) and tumor loads (**Figure 2D**). Notably, the tumor size distribution showed that tumors sizes were larger in the *Apc^min/+^Mlkl^-/-^*mice compared to the *Apc^min/+^* mice (**Figure 2E**). H&E staining also indicated that larger polyps were found in the intestines of the* Apc^min/+^Mlkl^-/-^*mice than in *Apc^min/+^* littermate controls (**Figure 2F**). The *Apc^min/+^*mice develop anemia and thymus atrophy, which is associated with severe intestinal tumorigenesis 24. We found that anemia and thymus atrophy were significantly exacerbated in *Apc^min/+^Mlkl^-/-^* mice compared to *Apc^min/+^*littermate controls, suggesting that loss of MLKL increased intestinal tumor development (**Figure 2G, 2H**). These results indicate that MLKL is dispensable for intestinal homeostasis under physiological condition and plays a protective role against intestinal tumorigenesis.

To investigate the mechanism by which MLKL deficiency promoted intestinal tumorigenesis in *Apc^min/+^Mlkl^-/-^* mice, we examined cell proliferation by staining proliferation marker Ki-67. The results revealed that the level of Ki-67 in the *Apc^min/+^Mlkl^-/-^*colonic crypts was significantly higher than in the *Apc^min/+^*mice, while no significant difference was found in WT and *Mlkl^-/-^* mice (**Figure 2I**). The observation of increased proliferation in the *Apc^min/+^Mlkl^-/-^*mice was further supported by western blotting. Compared with the *Apc^min/+^*mice, the *Apc^min/+^Mlkl^-/-^*mice exhibited increased levels of PCNA and decreased levels of cleaved caspase-3 (**Figure 2J**). Similar results were observed in colonic crypts from the *Apc^min/+^Mlkl^-/-^*intestinal tumors (**Figure 2K**). These results suggest that loss of MLKL accelerates intestinal tumorigenesis in *Apc^min/+^*mice by promoting proliferation and preventing apoptosis.

### Loss of MLKL enhances the activation of STAT3

**Figure 3:**
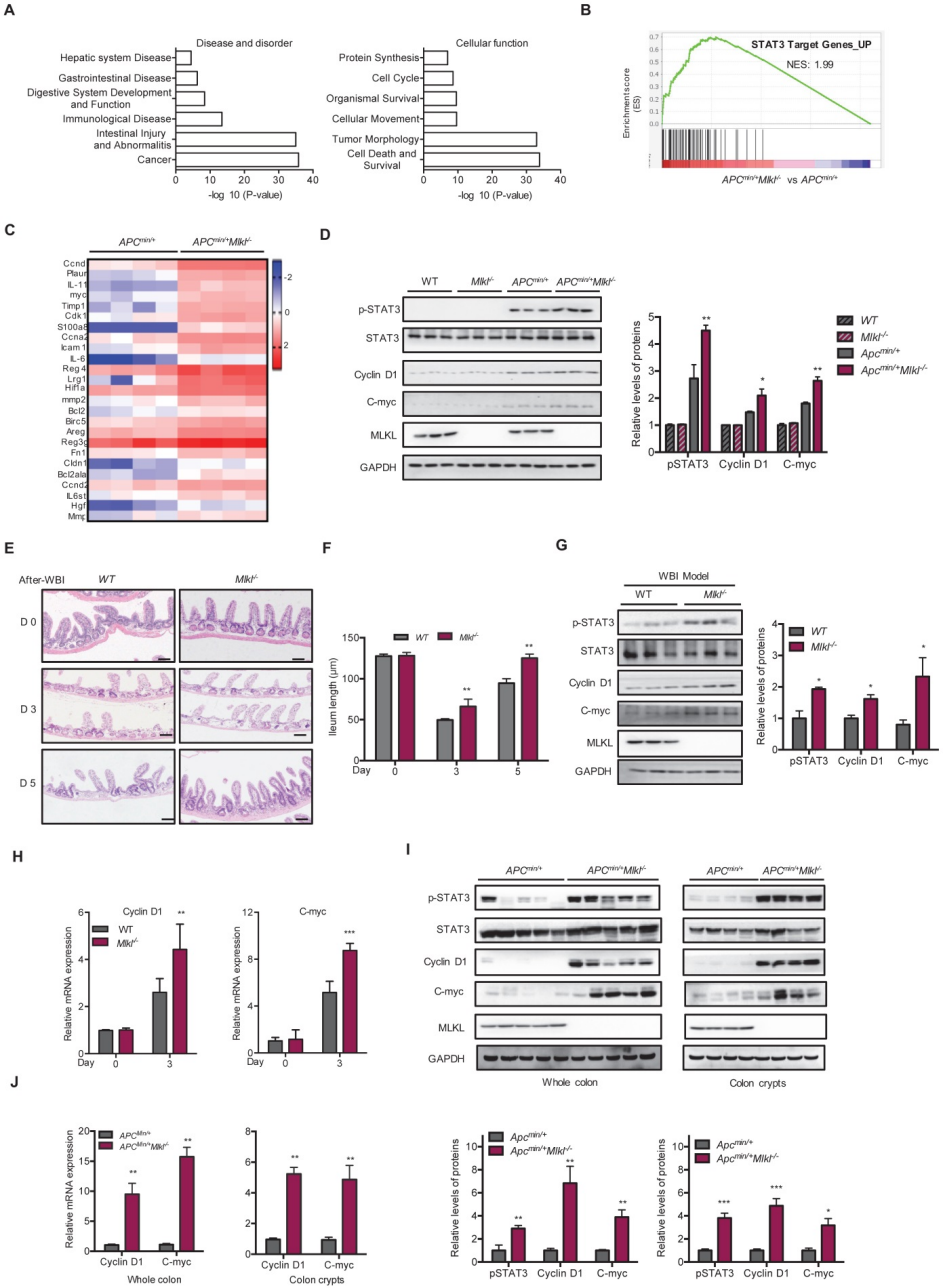
** Loss of MLKL enhances the activation of STAT3. (A)** Ingenuity Pathway Analysis (IPA) of the disease processes and cellular functions in 6-week-old* Apc^min/+^* and *Apc^min/+^Mlkl^-/-^* mice.** (B)** GSEA demonstrated enrichment of STAT3 target genes. **(C)** Heat map showing a summary of the expression of STAT3 pathway target genes in 6-week-old* Apc^min/+^* and *Apc^min/+^Mlkl^-/-^* mice. **(D)** Proteins isolated from 6-week-old* Apc^min/+^* and *Apc^min/+^Mlkl^-/-^* mouse intestines were analyzed by western blotting to assess STAT3 activation. **(E)** Images of H&E-stained intestinal tissues in 3 and 5 days after WBI. **(F)** Villus length in WT and *Mlkl^-/-^* intestines were measured after WBI. **(G)** Proteins isolated from WT and *Mlkl^-/-^* mice during regeneration were analyzed by western blotting to assess STAT3 activation. **(H)** Gene expression of intestinal tissues during regeneration (days 0 and 3) in WT and *Mlkl^-/-^* mice. **(I-J)** Whole intestine tissues and colonic crypts from 16-week-old *Apc^min/+^*and *Apc^min/+^Mlkl^-/-^* mice were lysed and measured the levels of Cyclin D1 and C-myc. Scale bars: 50 μm. * *p* < 0.05, ** *p* < 0.01, *** *p* < 0.001 *versus Apc^min/+^* mice.

To elucidate the mechanisms underlying loss of MLKL promoted intestinal tumorigenesis, microassay analysis was performed on the intestines of 6-week-old *Apc^min/+^*and *Apc^min/+^Mlkl^-/-^*mice. Because the *Apc^min/+^Mlkl^-/-^*mice showed similar histology compared to the *Apc^min/+^*mice at this time point, these gene alterations might be related to earlier events in intestinal tumorigenesis. A total of 5178 genes were upregulated and 1611 genes were downregulated in the *Apc^min/+^Mlkl^-/-^*intestines compared to *Apc^min/+^*intestines (**Figure S3A, S3B**). Ingenuity Pathway Analysis (IPA) showed that significantly impacted disease processes, including cancer, intestinal injury, abnormalities and intestinal disease (**Figure 3A**). Moreover, cell death, survival and tumor morphology were enriched in the *Apc^min/+^Mlkl^-/-^*mice (**Figure 3A**). To elucidate the MLKL-mediated signaling pathway, the upregulated genes were further functionally enriched by gene set enrichment analysis (GSEA) 40. The results indicated that the STAT3 signaling pathway-related gene set was enriched in *Apc^min/+^Mlkl^-/-^*mice intestines (**Figure 3B**). Further analysis of the genes related to STAT3 signaling indicated that the loss of MLKL significantly increased the levels of STAT3 target genes (**Figure 3C**). Moreover, phosphorylation of STAT3 (pSTAT3) was enhanced in the intestines of the 6-week-old* Apc^min/+^Mlkl^-/-^*mice compared to those of the *Apc^min/+^*mice, but no remarkable increase in expression of pSTAT3 was observed in either the intestines of the WT or *Mlkl^-/-^*mice (**Figure 3D**). The expression of STAT3 target genes, *Cyclin D1* and *C-myc,* was upregulated in the intestines of the *Apc^min/+^Mlkl^-/-^*mice compared to *Apc^min/+^*mice (**Figure 3D**).

Enhancement of STAT3 activation promotes intestinal regeneration during tissue injury and damage 41. We established whole-body irradiation (WBI)-induced intestinal injury and regeneration models to confirm the function of MLKL. Compared to that in the WT mice, the length of the villi in the *Mlkl^-/-^* mice was quickly recovered on days 3 and 5 after irradiation (**Figure 3E, 3F**), suggesting that MLKL deficiency accelerated intestinal regeneration process. There were higher protein levels of pSTAT3 in the *Mlkl^-/-^* intestines in the regeneration models (**Figure 3G**). The expression of *Cyclin D1* and *C-myc* was upregulated in the intestines of the *Mlkl^-/-^* mice compared to those of the WT mice in the WBI-induced intestinal regeneration models (**Figure 3G, 3H**). In addition, the levels of the progenitor cell-associated genes *Cd44* and *Sox9* was also significantly increased (**Figure S3C**). To determine whether *Apc^min/+^Mlkl^-/-^*mice exhibited higher regenerative potential during intestinal regeneration, we also performed WBI-induced regeneration experiments in *Apc^min/+^*and *Apc^min/+^Mlkl^-/-^*mice. By day 3 after irradiation, the protein levels of pSTAT3 were enhanced in the intestines of* Apc^min/+^Mlkl^-/-^*mice compared to the *Apc^min/+^*mice in the WBI-induced regeneration models (**Figure S3D**). Consistent with these findings, intestines of *Apc^min/+^Mlkl^-/-^*mice exhibited higher levels of Cyclin D1 and C-myc compared with the intestines of *Apc^min/+^*mice (**Figure S3D**).

To further confirm the role of MLKL on STAT3 activation during intestinal tumorigenesis, we examined STAT3 activation in 16-week-old *Apc^min/+^Mlkl^-/-^* and *Apc^min/+^* intestinal tumors. Western blotting revealed that the protein level of pSTAT3 were markedly increased in the intestinal tumors of the *Apc^min/+^Mlkl^-/-^*mice compared with those of the *Apc^min/+^* mice (**Figure 3I**). In addition, higher levels of pSTAT3 were also observed in the *Apc^min/+^Mlkl^-/-^*intestinal crypts (**Figure 3I**). Notably, protein expression of the STAT3 target genes Cyclin D1 and C-myc was increased in the intestines and crypts of the *Apc^min/+^Mlkl^-/-^*mice (**Figure 3I**). Consistently, qRT-PCR results indicated that the mRNA levels of the *Cyclin D1* and *C-myc* were significantly upregulated in the *Apc^min/+^Mlkl^-/-^*mice compared to the *Apc^min/+^* mice (**Figure 3J**). Similar to the *Apc^min/+^*mouse model, the expression levels of pSTAT3, Cyclin D1 and C-myc were greatly elevated in the *Mlkl^-/-^*→*Mlkl^-/-^* and WT→*Mlkl^-/-^* intestinal polyps compared with the *Mlkl^-/-^*→WT and WT→WT intestinal polyps at day 80 of the AOM/DSS-induced colitis-associated tumorigenesis (**Figure S3E**). Collectively, our results indicate that loss of MLKL promotes STAT3 activation in intestinal regeneration and tumorigenesis.

### MLKL deficiency exacerbates IL-6/STAT3 activation by promoting JAK2 phosphorylation

**Figure 4:**
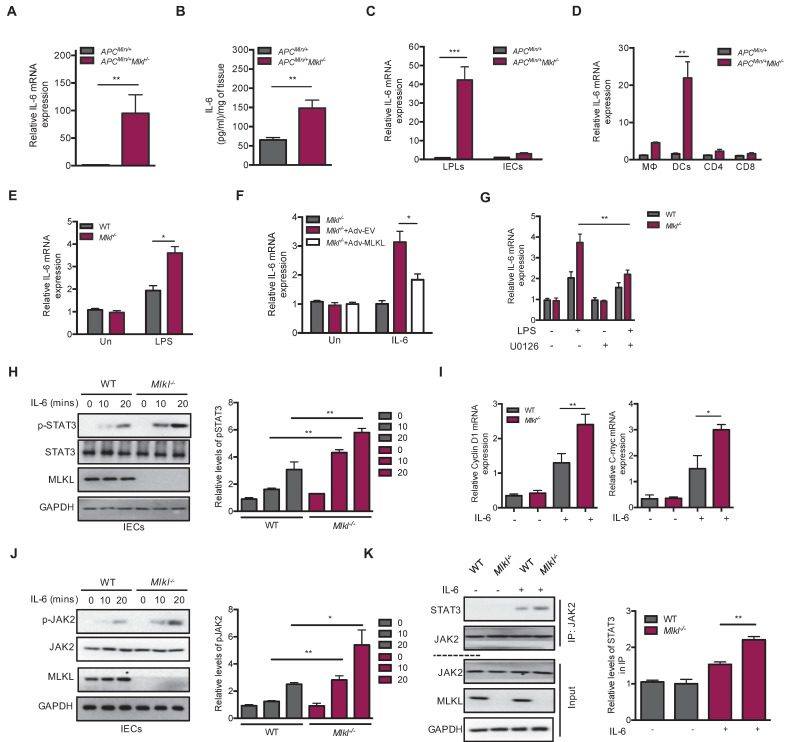
**MLKL deficiency exacerbates IL-6/STAT3 activation by promoting JAK2 phosphorylation. (A)** Quantitative analysis of IL-6 mRNA expression in *Apc^min/+^*and *Apc^min/+^Mlkl^-/-^*intestinal tumors. **(B)** The protein levels of IL-6 in *Apc^min/+^*and *Apc^min/+^Mlkl^-/-^*intestinal tumors as determined by ELISA. **(C)** Quantitative mRNA expression of IL-6 in isolated laminar propria lymphocytes (LPLs) and intestinal epithelial cells (IECs) of intestinal tumors from *Apc^min/+^*and *Apc^min/+^Mlkl^-/-^* mice. **(D)** Macrophages, dendritic cells, and T cells isolated by flow cytometric cell sorting were analyzed to assess IL-6 mRNA expression by qRT-PCR. **(E)** LPS-stimulated WT and *Mlkl^-/-^* BMDCs were analyzed for quantitative mRNA expression of IL-6 by qRT-PCR (LPS, 100 ng/ml). **(F)*** Mlkl^-/-^* BMDCs were overexpressed with MLKL for 48 h, cells then were stimulated with LPS and analyzed the expression of IL-6 mRNA. **(G)** WT and *Mlkl^-/-^* BMDCs were pre-treated 10 μM ERK inhibitor U0126 for 2 h, and then cells then were stimulated with LPS and analyzed the expression of IL-6 mRNA by qRT-PCR. **(H-I)** Primary WT and *Mlkl^-/-^*IECs isolated from 3-week-old mice were stimulated with 20 ng/mL IL-6. Cell lysates were analyzed for pSTAT3, Cyclin D1 and C-myc. **(J)** WT and *Mlkl^-/-^*IECs were stimulated with 20 ng/mL IL-6. Cell lysates were analyzed for pJAK2 (Y1007/Y1008). **(K)** The effect of MLKL on the interaction of JAK2 with STAT3. WT and *Mlkl^-/-^*IECs were stimulated with 20 ng/mL IL-6. Cell lysates were subjected to immunoprecipitation and western blotting with indicated antibody. * *p* < 0.05, ** *p* < 0.01, *** *p* < 0.001.

STAT3 activation is known to be required for IL-6 production, and IL-6/STAT3 signaling exerts a tumor-promoting function in intestinal tumorigenesis 18, 20. We found higher levels of IL-6 in the *Apc^min/+^Mlkl^-/-^* intestinal tumors than in those of the *Apc^min/+^* mice (**Figure 4A**). ELISA analysis further found higher levels of IL-6 protein in the *Apc^min/+^Mlkl^-/-^* intestinal tumors (**Figure 4B**). To explore the cellular source of this increased IL-6, we separated laminar propria lymphocytes (LPLs) and intestinal epithelial cells (IECs) from the intestinal tumors of *Apc^min/+^Mlkl^-/-^* and *Apc^min/+^* mice. The results showed that IL-6 was specifically upregulated in LPLs, but not in IECs (**Figure 4C**). Previous studies have shown that intestinal dendritic cells are responsible for IL-6 production 20, 42. We thus isolated certain types of cells from LPLs in *Apc^min/+^Mlkl^-/-^* and *Apc^min/+^* intestinal tumors. qRT-PCR results indicated that dendritic cells were responsible for IL-6 production (**Figure 4D**).

Our previous study revealed that MLKL deficiency promoted inflammatory responses via activating of ERK signaling in dendritic cells by 13. We thus explored whether dendritic cells produced IL-6 *in vitro* and found that the level of IL-6 was higher in LPS-stimulated *Mlkl^-/-^* bone marrow-derived dendritic cells (BMDCs) (**Figure 4E**). In contrast, MLKL overexpression partially decreased LPS-induced IL-6 expression in the *Mlkl^-/-^* BMDCs (**Figure 4F**). To address the role of MEK/ERK activation in IL-6 production in *Mlkl^-/-^* BMDCs, the MEK/ERK inhibitor U0126 was used. U0126 blocked the LPS-induced IL-6 expression in the *Mlkl^-/-^* BMDCs (**Figure 4G**). Together, these data suggest that IL-6 is overproduced in MLKL-deficient dendritic cells through the activation of ERK signaling.

To dissect IL-6-mediated hyperactivation of STAT3 in *Mlkl^-/-^* epithelial cells, we isolated primary WT and *Mlkl^-/-^* IECs and stimulated them with extracellular IL-6. The results showed that the levels of p-STAT3 were higher in the *Mlkl^-/-^* IECs than in the WT IECs following treatment with IL-6 (**Figure 4H**). Similarly, higher expression levels of the *Cyclin D1* and *C-myc* were observed in *Mlkl^-/-^* IECs (**Figure 4I**). Moreover, the IL-6-induced increase in pSTAT3 in the context of knocked down MLKL expression was also found in colorectal carcinoma HT-29 cells (**Figure S4**). Accumulating evidence suggests that phosphorylation and activation of JAK2 play a critical role in IL-6/STAT3 activation 23. To explore whether MLKL interfered with the activation of JAK2, we assessed the levels of JAK2 phosphorylation in the *Mlkl^-/-^* and WT IECs following treatment with IL-6. As shown in **Figure 4J**, MLKL deficiency increased IL-6-induced JAK2 phosphorylation. Of note, co-immunoprecipitation experiments revealed that the interaction JAK2 and STAT3 was increased in *Mlkl^-/-^* IECs compared to WT IECs (**Figure 4K**). These data together indicate that MLKL deficiency promotes intestinal tumorigenesis through activation of the IL-6/JAK2/STAT3 axis.

### Blocking IL-6 signaling inhibits intestinal tumorigenesis in *Apc^min/+^Mlkl^-/-^* mice

**Figure 5:**
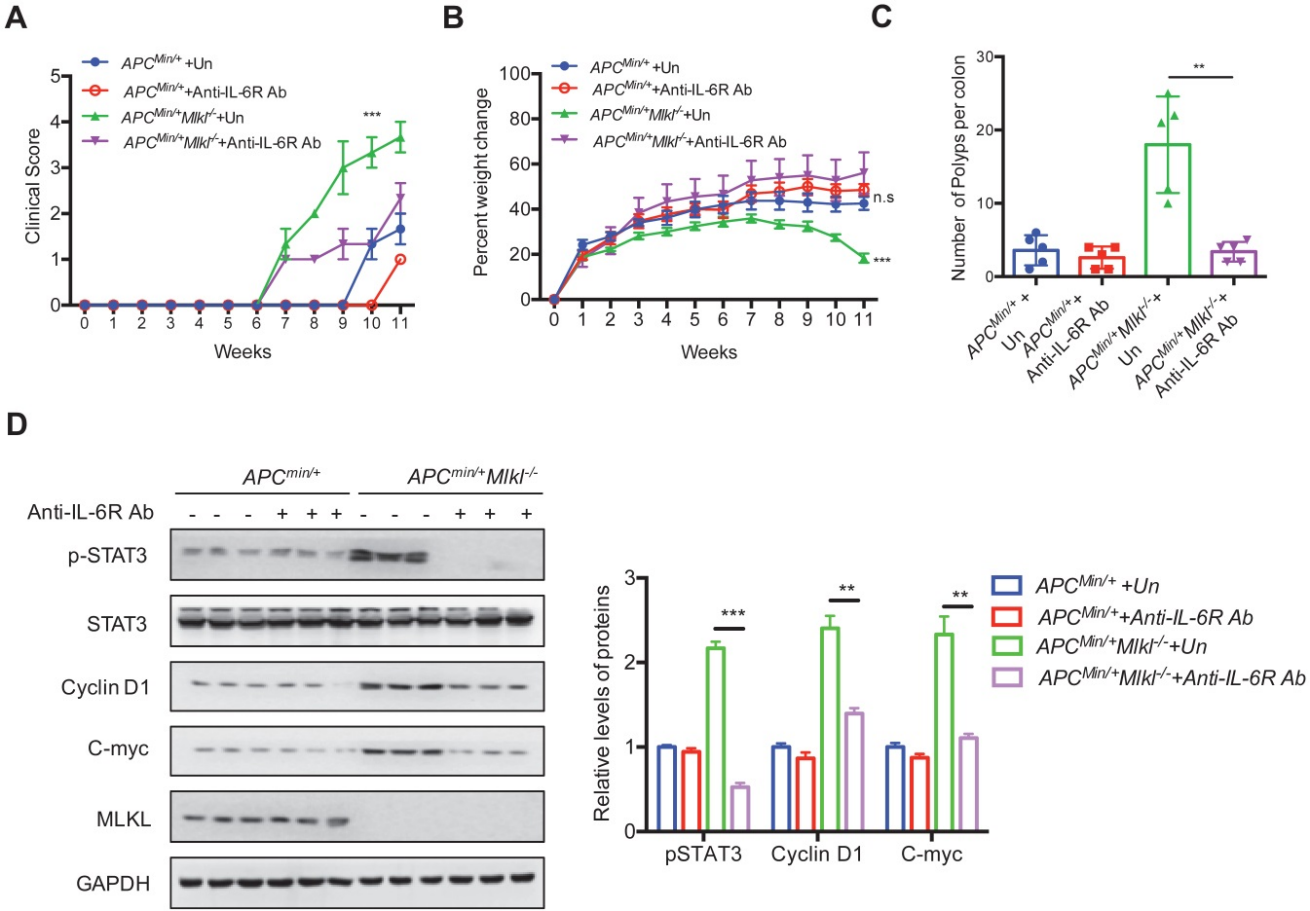
**Blocking IL-6 signaling inhibits intestinal tumorigenesis in *Apc^min/+^Mlkl^-/-^* mice. (A and B)** 4-week-old *Apc^min/+^* and *Apc^min/+^Mlkl^-/-^* mice were administrated with anti-IL6R antibody weekly for 10 weeks. Clinical score and the percent weight change were monitored. **(C)** Quantification of polyp formation in PBS- or anti-IL6R-treated mice as indicated.** (D)** Intestinal tissues were harvested from PBS-treated *Apc^min/+^Mlkl^-/-^* and anti-IL6R-treated *Apc^min/+^Mlkl^-/-^* mice and then lysed. The levels of pSTAT3, Cyclin D1 and C-myc were determined by western blotting. ** *p* < 0.01 and *** *p* < 0.001.

The above observations suggest that loss of MLKL leads to hyperactivation of IL-6 signaling. Next, we sought to explore whether the IL-6/STAT3 axis contributed to the increase in intestinal tumors in *Apc^min/+^Mlkl^-/-^*mice. To this end, we used IL-6 receptor antibodies (anti-IL-6R) to block IL-6 signaling. *Apc^min/+^* and *Apc^min/+^Mlkl^-/-^* mice were intraperitoneally injected with anti-IL-6R weekly for 10 weeks. The anti-IL6R-treated *Apc^min/+^Mlkl^-/-^* mice exhibited a lower clinical score and significant weight gain compared to the untreated (UT) *Apc^min/+^Mlkl^-/-^* mice (**Figure 5A, 5B**). However, the anti-IL-6R-treated *Apc^min/+^* mice exhibited mild changes in clinical score and body weight compared to the UT *Apc^min/+^* mice (**Figure 5A, 5B**). After IL-6R antibody treatment for 10 weeks, the anti-IL6R-treated *Apc^min/+^Mlkl^-/-^*mice exhibited fewer intestinal polyps than the UT *Apc^min/+^Mlkl^-/-^*group (**Figure 5C and Figure S5A**). Consistent with the decreased intestinal polyps in the anti-IL-6R-treated *Apc^min/+^Mlkl^-/-^* mice, the anemia and thymus atrophy in the *Apc^min/+^Mlkl^-/-^* mice were considerably alleviated after anti-IL-6R treatment (**Figure S5B, S5C**). Western blotting showed that the expression levels of pSTAT3, Cyclin D1 and C-myc was significantly suppressed in the anti-IL-6R-treated *Apc^min/+^Mlkl^-/-^* mice compared to the UT *Apc^min/+^Mlkl^-/-^* mice (**Figure 5D**). Thus, these results demonstrate that IL-6/STAT3 hyperactivation in *Apc^min/+^Mlkl^-/-^* mice plays a causative role in increased intestinal polyps formation.

### The levels of MLKL has prognostic implications in colorectal cancer

**Figure 6:**
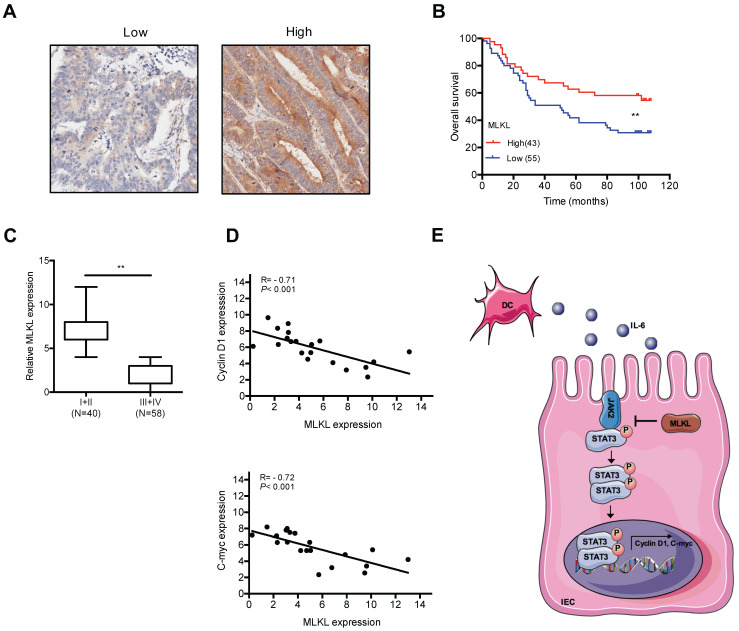
**The levels of MLKL has prognostic implications in colorectal cancer. (A-B)** Expression of MLKL in colorectal cancer samples detected by IHC. Overall survival of patients based on MLKL expression levels conducted by Kaplan-Meier plot. **(C)** Boxed plot of MLKL expression assessed at different clinical stages. **(D)** Pearson correlation analysis of MLKL and *Cyclin D1* (P < 0.001; R = - 0.71) and MLKL and *C-myc* (P < 0.001; R = - 0.72) in human colorectal cancer. **(E)** Model of MLKL negatively impacts IL-6/STAT3 signaling in intestinal tumorigenesis. ** *p* < 0.01.

To examine the potential role of MLKL in human colorectal tumors, we analyzed MLKL expression colorectal tumor samples. Notably, Kaplan-Meier survival analysis indicated that low MLKL expression exhibited shorter overall survival than patients with high MLKL expression (**Figure 6A, 6B**). Moreover, the expression of MLKL was inversely linked to the stages of disease progression in colorectal tumors (**Figure 6C**). Given our findings that loss of MLKL increased activation of STAT3 in spontaneous intestinal tumorigenesis, we further examined the potential correlation between MLKL expression and STAT3 activation in human colorectal tumor samples. The levels of MLKL mRNA are negatively correlated with *Cyclin D1* and *C-myc* expression (**Figure 6D**)*.* These clinical findings indicate that low MLKL expression level may be prognostic for the malignant progression of colorectal cancer.

## Discussion

In this study, we showed that loss of MLKL activated the STAT3 signaling pathway to accelerate intestinal regeneration and tumorigenesis. Mechanismly, we found that loss of MLKL increased the phosphorylation of JAK2 and promoted the association of JAK2 with STAT3, leading to hyperactivation of STAT3. Furthermore, IL-6 was upregulated in MLKL-deficient dendritic cells via increased ERK activation. In summary, MLKL exhibits a suppressive effect in the progression of intestinal tumors by regulating the IL-6/JAK2/STAT3 axis (**Figure 6E**).

MLKL is a major necroptosis regulatory protein that is widely expressed in tissues and organs 6, 36-37. Increasing evidences suggest that MLKL is involved in pathophysiological conditions, including liver injury, insulin resistance and T2D, cholestasis, and hepatitis 43-46. Experimental studies demonstrate that MLKL protects mice from against mucosal infection and promotes mucosal repair 13, 47. Several recent reports have indicated that the prognostic value of MLKL protein expression in multiple malignant tumors 8-9. We previously reported that the loss of MLKL led to increased susceptibility to colitis-associated tumorigenesis 13. Herein, we demonstrated that loss of MLKL promoted intestinal tumor progression and increased tumor burden. Our results highlighted that the loss of MLKL activated the STAT3 signaling pathway. Moreover, in the absence of MLKL, high levels of phosphorylated STAT3 were found in intestinal regeneration and tumorigenesis. Sustained STAT3 activation contributed to tumor progression, aberrant JAK2/STAT3 has been detected in a variety of tumor types, suggesting that STAT3 exhibits promise as a drug target for cancer therapeutics 48. We found that loss of MLKL strengthened the association of JAK2 with STAT3, thereby promoting STAT3 phosphorylation and intestinal tumorigenesis. Our study provides new insights into the role of MLKL serves as a negative regulator in intestinal tumorigenesis through suppressing STAT3 activation.

Several recent reports indicated that the lower levels of MLKL were correlated with poor overall survival in malignant tumors 12, 49-52. Notably, we observed that reduced MLKL expression in patients with colorectal tumors was correlated with high histological grade and decreased overall survival. Furthermore, we saw that there was a negative relationship between MLKL and the STAT3 target genes expression in colorectal tumors. In agreement, we also found that the size of tumors in the *Apc^min/+^Mlkl^-/-^*mice were larger than those in the *Apc^min/+^*mice. These data suggested that MLKL plays a tumor-suppressive role during intestinal tumorigenesis.

IL-6 forms a complex with the IL-6 receptor, leading to persistent activation of STAT3 53. The levels of IL-6 are dramatically increased in serum and tumor samples of humans and mice with colorectal cancer, and high IL-6 levels in colorectal cancers are found to correlate with poor prognosis 54-55. IL-6 promotes the survival of colon carcinoma cells *in vitro*; however blocking IL-6 signal prevents tumor growth 56-57. *Mlkl^-/-^*mice exhibited massive inflammatory responses and exhibited high IL-6 expression during colitis and colitis-associated tumorigenesis 13. Herein, we found that IL-6 was elevated in the *Apc^min/+^Mlkl^-/-^* intestine tumors. Further experiments demonstrated that immune cells, especially dendritic cells, were the main IL-6-producing cells in the *Apc^min/+^Mlkl^-/-^* intestines. Interestingly, the activation of ERK pathway promoted IL-6 expression in *Mlkl^-/-^*dendritic cells, indicating that MLKL deficiency induces IL-6 production in dendritic cells by activating ERK. However, we cannot exclude the possibility that MLKL is involved in other cell types. The contribution of the cell-specific function of MLKL to the suppression of intestinal tumors needs to be further investigated using MLKL conditional knockout mice in future studies.

In summary, our data reveal that MLKL exhibit a suppressive effect during intestinal tumorigenesis via suppressing IL-6/STAT3 signaling pathway. Our results also highlight that interfering with IL-6/STAT3 axis could be a better therapeutic option for intestinal tumor patients with low MLKL expression.

## Supplementary Material

Supplementary figures and tables.

## Abbreviations

AOM: Azoxymethane
BMDCs: Bone marrow-derived dendritic cells
DAI: Disease activity index
DEGs: Differentially expressed genes
DSS: Dextran sodium sulfate
FDR: False Discovery Rate
IECs: Intestinal epithelial cells
IL-6: Interleukin-6
IL-6R: Interleukin-6 receptor
JAK2: Janus kinase 2
LPLs: Laminar propria lymphocytes
MLKL: Mixed lineage kinase domain-like protein
PCNA: Proliferating cell nuclear antigen
qRT-PCR: Quantitative reverse-transcriptase PCR
RHIMs: RIP homotypic interaction motifs
RIPK1: Receptor-interacting protein kinase 1
STAT3: Signal transducer and activator of transcription 3
UT: Untreated
WBI: Whole-body irradiation
