# Supplementary material for: MLKL inhibits intestinal tumorigenesis by suppressing STAT3 signaling pathway
Source: Int J Biol Sci. 2021 Feb 17;17(3):869–81. doi: 10.7150/ijbs.56152 (PMC7975698; doi:10.7150/ijbs.56152)
Supplement: Supplementary file 1 — Supplementary figures and tables. [file ijbsv17p0869s1.pdf]

Appendix A. Supplementary material

Title: MLKL inhibits intestinal tumorigenesis by suppressing STAT3 signaling pathway

Qun Zhao [1,3 ✉], Xinran Cheng [1], Jian Guo [1], Yun Bi [1], Li Kuang [2], Jianhua Ren [2], Jing Zhong [1], Longrui Pan [1], Xudong Zhang [1], Yang Guo [1], Yongqiang Liu [4], Shu Jin [5], Yan Tan [1], Xianjun Yu [1, ✉]

Supplemental Data

Figure S1, Related to Figure 1

Figure S2, Related to Figure 2

Figure S3, Related to Figure 3

Figure S4, Related to Figure 4

Figure S5, Related to Figure 5

Supplemental figures and legends

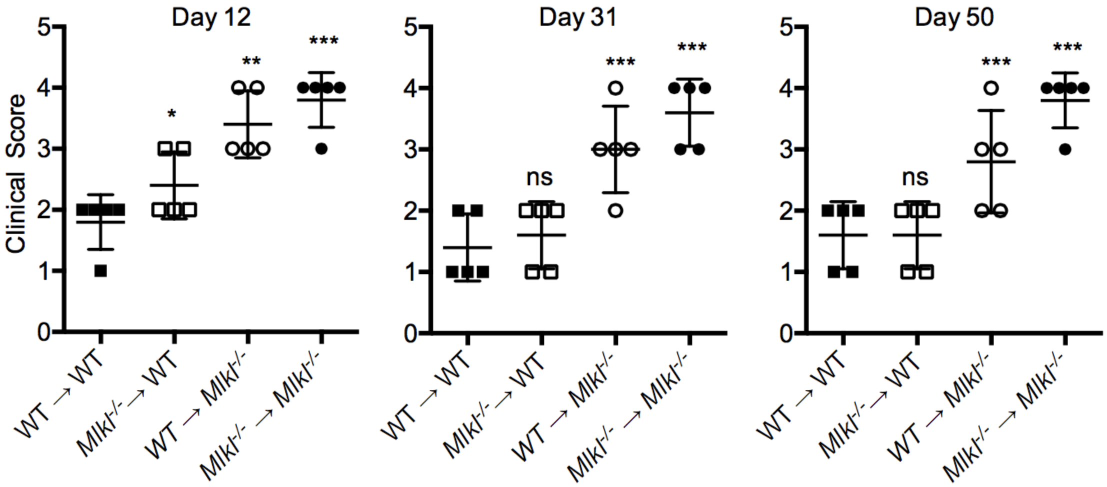

Figure S1, Related to Figure 1. Clinical scores of disease from the chimeric mice in the AOM/DSS models were calculated at days 12, 31 and 50. * $p < 0.05$, ** $p < 0.01$ and *** $p < 0.001$ *versus* WT → WT groups.

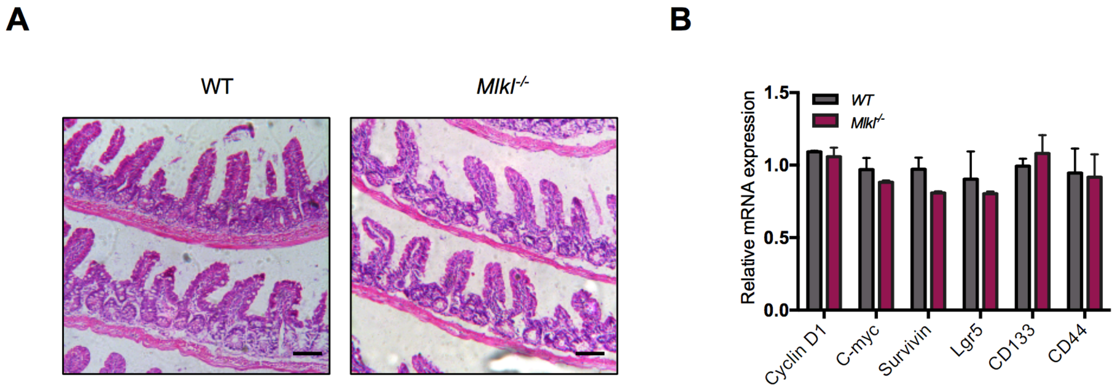

Figure S2, Related to Figure 2. MLKL is dispensable for the self-renewal under physiological conditions

(A) H&E staining of representative intestines from 12-month-old WT and *Mlkl*$^{-/-}$ mice. (B) qRT-PCR analysis of gene expression in the intestines of WT and *Mlkl*$^{-/-}$ mice as indicated.

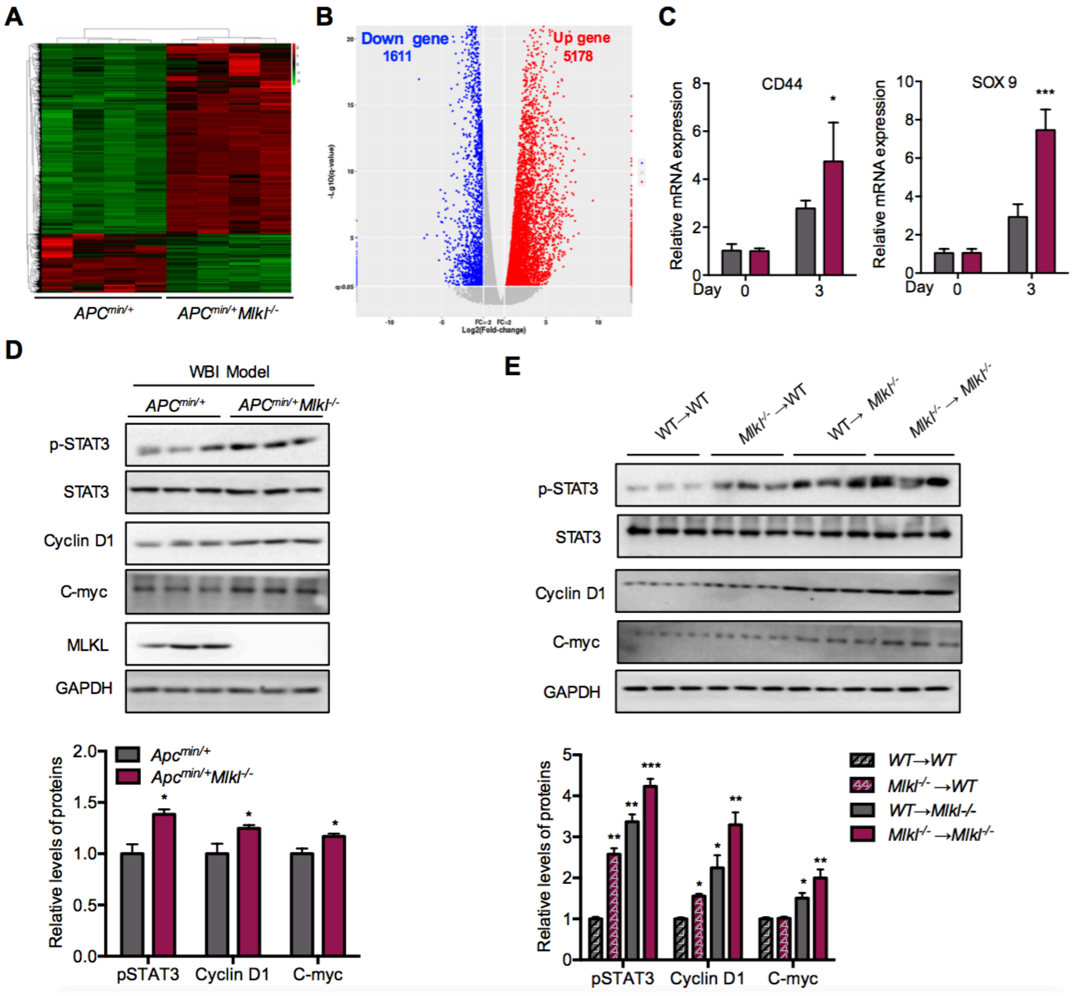

Figure S3, Related to Figure 3. Loss of MLKL stimulates STAT3 signaling

(A) Heat map analysis of differentially mRNA in 6-week-old *Apc*$^{min/+}$ mice and *Apc*$^{min/+}$*Mlkl*$^{-/-}$ intestinal tissues. (B) Upregulated and downregulated genes in intestinal tissues of *Apc*$^{min/+}$ mice and *Apc*$^{min/+}$*Mlkl*$^{-/-}$ mice was shown. (C) The expression of CD44 and SOX 9 the intestine of WT and *Mlkl*$^{-/-}$ mice during regeneration (days 0 and 3). (D) The expression of pSTAT3 and STAT3 target gene in intestinal tissues during regeneration (days 0 and 3) in *Apc*$^{min/+}$ and *Apc*$^{min/+}$*Mlkl*$^{-/-}$ mice. (E) Protein lysates were isolated from intestine polyps from four groups of AOM/DSS-treated chimeric mice. Lysates were analyzed by western blotting to detect the expression of pSTAT3 and STAT3 target genes. * $p < 0.05$, ** $p < 0.01$ and *** $p < 0.001$.

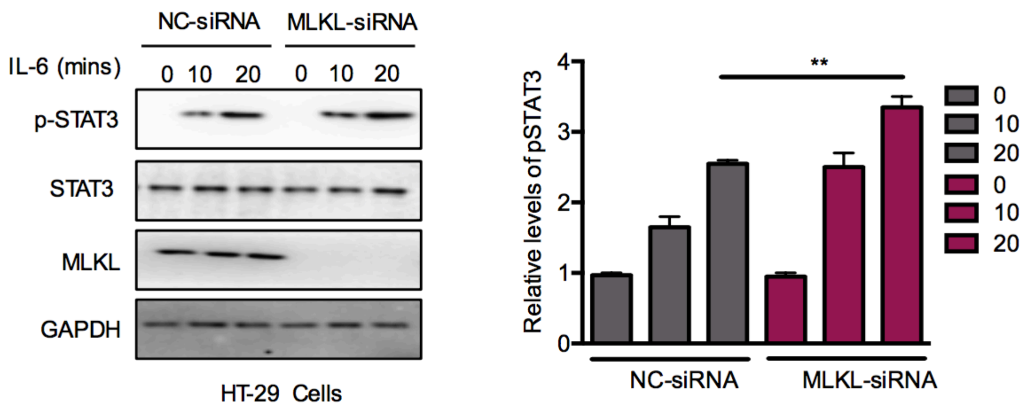

Figure S4, Related to Figure 4. MLKL deficiency exacerbates IL-6/STAT3 activation HT-29 cells were treated with IL-6 for the indicated time interval in which MLKL was knocked down. Cell lysates collected at indicated time points were analyzed for pSTAT3. * $p < 0.05$.

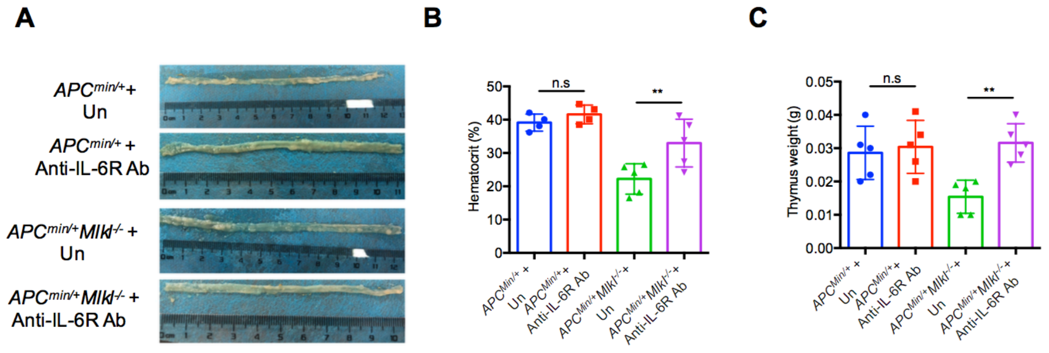

Figure S5, Related to Figure 5. Blocking IL-6 signaling suppresses intestinal tumorigenesis

(A) Images of colons isolated from $Apc^{min/+}$ and $Apc^{min/+}Mlkl^{-/-}$ animals after 10 weeks of anti-IL6R therapy. (B-C) Hematocrit (B) and thymus weight (C) of $Apc^{min/+}$ and $Apc^{min/+}Mlkl^{-/-}$ mice after anti-IL6R therapy for 10 weeks. ** $p < 0.01$.

Supplemental tables

Table S1. Related to Fig. 1. The stool and bleeding score

**Group**	**Day 12**					**Day 31**					**Day 50**				
WT→ WT	2	2	2	2	1	1	2	1	1	2	1	1	2	2	2
Mlkl$^{-/-}$→ WT	2	3	3	2	2	1	2	2	1	2	1	1	2	2	2
WT→ *Mlkl*$^{-/-}$	4	3	4	3	3	3	3	4	2	3	3	2	2	3	4
Mlkl$^{-/-}$→ *Mlkl*$^{-/-}$	4	4	3	4	4	4	4	3	4	3	4	4	3	4	4
